# Difference in vaginal microecology, local immunity and HPV infection among childbearing-age women with different degrees of cervical lesions in Inner Mongolia

**DOI:** 10.1186/s12905-019-0806-2

**Published:** 2019-08-12

**Authors:** Jing-Jing Zheng, Jing-Hui Song, Cong-Xiang Yu, Fei Wang, Peng-Cheng Wang, Jing-Wei Meng

**Affiliations:** 1 Department of Obstetrics and Gynecology, The Affiliated Hospital of Inner Mongolia Medical University, No. 1 of TongDao North Street, HuiMin District, Huhhot, 010059 Inner Mongolia China; 2 Department of Obstetrics and Gynecology, Inner Mongolia Maternal and Child Health Care Hospital, Huhhot, 010020 Inner Mongolia China

**Keywords:** Immune and HPV infection, Cervical lesions, Cervical squamous cell carcinoma, Cervical intraepithelial neoplasia, Pathogen infections

## Abstract

**Background:**

This study aims to investigate the difference in vaginal microecology, local immunity and HPV infection among childbearing-age women with different degrees of cervical lesions.

**Methods:**

A total of 432 patients were included in this study. Among these patients, 136 patients had LSIL, 263 patients had HSIL and 33 patients had CSCC. These patients were assigned as the research groups. In addition, 100 healthy females were enrolled and assigned as the control group.

**Results:**

The microbiological indexes of vaginal secretions were evaluated. Furthermore, the concentrations of SIgA, IgG, IL-2 and IL-10 in vaginal lavage fluid, as well as the presence of HPV, mycoplasma and Chlamydia in cervical secretions, were detected. The results is that: (1) Differences in evaluation indexes of vaginal microecology among all research groups and the control group were statistically significant (*P* < 0.0001). As the degree of cervical lesions increased, the number of Lactobacillus decreased, and there was an increase in prevalence of bacterial imbalance, and the diversity, density and normal proportion of bacteria was reduced. Furthermore, the incidence of HPV, trichomonads, clue cell and Chlamydia infection increased. Moreover, the positive rate of H_2_O_2_ decreased, while the positive rates of SNa and GADP increased. (2) Differences in the ratio of IL-2 and IL-10 in the female genital tract among all research groups and the control group were statistically significant (*P* < 0.0001).

**Conclusions:**

As the degree of cervical lesions increased, IL-2 decreased, IL-10 increased and IL-2/IL-10 decreased, while SIgA and IgG were elevated. The reduction of dominant Lactobacillus in the vagina, impairment of H_2_O_2_ function, flora ratio imbalance, pathogen infections, reduction in IL-2/IL-10 ratio, and changes in SIgA and IgG levels could all be potential factors that influenced the pathogenicity of HPV infection and the occurrence and development of cervical lesions.

## Background

Studies have revealed that high-risk human papillomavirus (HPV) infection is the major cause of cervical lesions [1, 2], and that 80% of women have been infected with HPV in their lifetime, but only a small number of these population have developed cervical lesions [3, 4]. In addition to HPV infection, other synergistic factors also affect the pathogenicity of HPV infections [5]. However, it remains to be determined whether local changes in the microecology and immunity of the vagina affect the pathogenicity and progression of HPV infection, since the cervix is exposed in the vagina. Therefore, in the present study, the microecological indicators of vaginal secretions of childbearing-age women with cervical intraepithelial neoplasia (CIN) and cervical squamous cell carcinoma (CSCC) were evaluated. Furthermore, the concentrations of immunoglobulin A (SIgA), immunoglobulin G (IgG), interleukin-2 (IL-2) and interleukin-10 (IL-10) in vaginal lavage fluid, as well as the incidence of HPV, mycoplasma and Chlamydia infection in vaginal secretions, were detected, in order to investigate the potential relationship among changes in vaginal microecology, local immune factor expression and HPV infection, and cervical lesions.

## Methods

### Subjects and study design

From November 2012 to September 2015, a total of 432 childbearing-age patients were enrolled into this study. Among these patients, 136 patients had low-grade squamous intraepethelial lesions (LSILs, CIN I; assigned as the LSIL group), 263 patients had high-grade squamous intraepethelial lesions (HSILs, CIN II or CIN III; assigned as the HSIL group), and 33 patients had cervical squamous cell carcinoma (CSCC, assigned as the CSCC group). These patients were generally assigned as the research group. In addition, 100 healthy (without intraepethelial lesions CSCC) women of childbearing-age were enrolled during the same period, and were assigned as the control group. The average age of all subjects was 42.22 ± 8.76 years old, and the age variances among all groups were homogeneous and comparable. The present study was approved by the Ethics Committee of the Affiliated Hospital of Inner Mongolia Medical University, China. All patients provided a signed informed consent.

The results of each test (as described below in detailed methods) were compared among each group. It was hypothesized that the degree of cervical lesions was correlated to the reduction of dominant Lactobacillus in the vagina, impairment of H_2_O_2_ function, flora ratio imbalance, pathogen infections, reduction in IL-2/IL-10 ratio, and changes in SIgA and IgG levels.

Sample size calculation was based on the rate of chlamydia infection and sIgA, and it was estimated to be at least 30 subjects in each group.

1. Inclusion criteria for all subjects: patients in childbearing age; patients with a history of sexual intercourse; patients who did not receive systemic antibiotics within 2 weeks; patients who did not receive medication of sexual hormones within 3 months; patients without vaginal lavage drug delivery within 1 week; patients who did not have sexual intercourse, or did not undergo tub bath, vaginal douching, or an operation within 24 h before visiting the doctor; patients without chemotherapy history before sampling.
2. Exclusion criteria for all subjects: women in pregnancy or lactation; patients with low immune function due to various autoimmune diseases, malignant tumors of other organs, medication of immune inhibitors, or other factors; patients combined with serious diseases in the heart, liver, kidney and hematopoietic system, as well as mental illness.

### Specimen acquisition

#### Vaginal secretion collection

(1) The secretion on the upper 1/3 segment of the vagina was collected by rotating three sterile long cotton swabs to evaluate the vaginal microecology and detect trichomonads, *Candida albicans*, clue cells and the five preformed enzymes. (2) The cervical secretion was obtained using a special brush to detect HPV, mycoplasma and Chlamydia. (3) The upper 1/3 segment of the vagina and exocervix were rinsed with 5 ml of normal saline, and lavage fluid was drawn from the posterior fornix. After centrifugation, the supernatant was obtained and preserved at − 20 °C to detect for immune factors.

#### Acquisition of cervical tissues

Under a colposcope, suspected cervical lesion tissues were obtained and sent to the Department of Pathology for pathological diagnosis.

### Laboratory tests

#### Evaluation of vaginal microecology

1. Microscopic detection after gram staining: (A) Flora density: under a 10 × 100 times microscope with oil immersion, observation results were recorded as grades I-IV (denoted as + to ++++), according to the average number of bacteria in each visual field. (B) Flora diversity: results were classified into grades I-IV (denoted as + to ++++), according to the species number of visible bacteria. (C) Dominant bacterium: Under a microscope, the microorganism species with the largest number was defined as the dominant bacterium.
2. Clue cells, trichomonads and *Candida albicans* were detected under a microscope by hematoxylin and eosin (H&E) staining.
3. Vaginal preformed enzymes: catalase (hydrogen peroxide, H_2_O_2_), neuraminidase (SNa), leukocyte esterase (LE), glucuronidase (GUS) and coagulase (GADP) were detected using Aerobic Vaginitis and Bacterial Vaginosis Diagnostic Strip Sets produced by Beijing Zhongsheng Jinyu Diagnostic Technology Co. Ltd.

#### Determination of vaginal local immune factors

SIgA, IgG, IL-2 and IL-10 levels in the supernatant were detected using corresponding enzyme-linked immunosorbent assay (ELISA) kits, according to manufacturer’s instructions; and the levels in the sample were calculated.

#### Detection of vaginal HPV, mycoplasma and chlamydia

After acquisition, the sample tube with the brush head was immediately sent to the Clinical Laboratory. HPV, mycoplasma and Chlamydia were detected by DNA testing.

### Diagnostic criteria for vaginal microecology

1. Flora density: “++” and “+++” were considered normal, while “+” and “++++” were considered abnormal;
2. Flora diversity: “++” and “+++” were considered normal, while “+” and “++++” were considered abnormal;
3. When the dominant bacterium was Lactobacillus, the flora was determined as a normal flora; when clue cells was (+), it was determined as bacterial vaginosis (BV); and when other pathogen infections occurred, the condition was determined as flora imbalance.
4. H_2_O_2_ ≥ 2 μmol/L was considered positive, SNa ≥7 U/L was considered positive, LE ≥9 U/L was considered positive, GUS ≥15 U/L was considered positive, and GADP ≥20 U/L was considered positive. When H_2_O_2_ was positive, it was determined as normal function of Lactobacillus; and when SNa, LE, GUS and GADP were all negative, it was determined as normal.

### Statistical analysis

Data were analyzed in the Public Health College of the Medical University of Tianjin using SAS 9.2 statistical software. Quantitative data were expressed as mean ± standard deviation (Mean ± SD). Qualitative data were described by absolute and relative indices. Quantitative data were analyzed by analysis of variance (ANOVA) or rank-sum test. Qualitative data were analyzed by Chi-square test or exact probability test. Statistical tests were conducted using the two-sided test. *P* ≤ 0.05 was considered statistically significant.

## Results

### HPV infection of the research groups and control group

Differences in HPV infection rate among the research groups and control group were statistically significant (*P* < 0.0001). High-risk HPV infection rate was as high as 90.91% in the CSCC group (Table 1).

**Table 1:** The types and infection rates of HPV in the study groups and the control group

The lesion group	High-risk type n (%)	Multiple infections n (%)	Low-risk type n (%)	Negative n (%)	*P*
CG	22 (22.00)	1 (1.00)	3 (3.00)	74 (74.00)	< 0.0001
LSIL	98 (72.06)	6 (4.41)	4 (2.94)	28 (20.59)	
HSIL	195 (74.14)	16 (6.08)	3 (1.14)	49 (18.63)	
CSCC	30 (90.91)	0 (0.00)	0 (0.00)	3 (9.09)	

### Evaluation of vaginal microecology in the research groups and control group

Differences in the evaluation indexes of vaginal microecology among all the research groups and the control group were statistically significant (*P* < 0.0001). As the degree of cervical lesions increased, the proportion of Lactobacilli decreased, the prevalence of bacterial imbalance increased, and the diversity, density and normal proportion of bacteria was reduced. Furthermore, the positive rate of H_2_O_2_ gradually decreased with the increase in the degree of lesions, which rebounded and increased in the CSCC group. The positive rate of SNa was the highest in the LSIL and CSCC groups, while the positive rates of LE, GUS and GADP were the highest in the CSCC group. The differences in the positive rates of H_2_O_2_, SNa and GADP among these groups were statistically significant (*P* < 0.05). The infection rates of pathogenic microorganisms trichomonads, BV and Chlamydia increased with the increase in the degree of cervical lesions. The difference in infection rates of BV, Chlamydia and *Candida albicans* among these groups were statistically significant (*P* < 0.05). In patients with HPV (+) in the control group, the positive rate of H_2_O_2_ and the density, diversity and normal proportion of flora were the highest, the rate of flora imbalance and the levels of positive LE, GUS and GADP were the lowest, while the infection rate of trichomonads, BV, mycoplasma and Chlamydia were the lowest; and differences among these groups were statistically significant (Tables 2 and 3).

**Table 2:** The proportion of the evaluation indexes of each vagina microecology in the study group and the control group

Groups	Dominant fungi			Flora diversity			Flora density			Advance into enzyme				
	G + bacillus (normal)	dysbacteriosis	G + short coli (BV)	+	++/+++	++++	+	++/+++	++++	H_2_O_2_positive	SNa positive	LE positive	GUS positive	GADP positive
CG	67 (67.00)	6 (6.00)	27 (27.00)	4 (4.00)	89 (89.00)	7 (7.00)	4 (4.00)	89 (89.00)	7 (7.00)	53 (53.00)	11 (11.00)	31 (31.00)	2 (2.00)	6 (6.00)
CG HPV(−)	50 (67.57)	6 (8.11)	18 (24.32)	4 (5.41)	64 (86.49)	6 (8.11)	4 (5.41)	64 (86.49)	6 (8.11)	38 (51.35)	8 (10.81)	23 (31.08)	2 (2.70)	5 (6.76)
CG HPV (+)	17 (65.38)	0 (0.00)	9 (34.62)	0 (0.00)	25 (96.15)	1 (3.85)	0 (0.00)	25 (96.15)	1 (3.85)	15 (57.69)	3 (11.54)	8 (30.77)	0 (0.00)	1 (3.85)
LSIL	67 (49.26)	10 (7.35)	59 (43.38)	8 (5.88)	119 (87.5)	9 (6.62)	8 (5.88)	119 (87.5)	9 (6.62)	45 (33.09)	35 (25.74)	60 (44.44)	8 (5.88)	19 (13.97)
HSIL	117 (44.49)	40 (15.21)	106 (40.3)	33 (12.55)	212 (80.6)	18 (6.84)	33 (12.55)	212 (80.61)	18 (6.84)	78 (29.66)	40 (15.21)	119 (45.25)	6 (2.28)	46 (17.49)
CSCC	7 (21.21)	20 (60.61)	6 (18.18)	11 (33.33)	19 (57.6)	3 (9.09)	11 (33.33)	19 (57.58)	3 (9.09)	17 (51.52)	8 (24.24)	18 (54.55)	3 (9.09)	9 (27.27)
X^2^	80.1951			27.926			27.9264							
*P*	<.0001			<.0001			<.0001			0.0001	0.0111	0.0737	0.0627	0.0057

Data are expressed as n (%)

**Table 3:** The proportion of pathogenic microorganism infection in the study group and the control group

Group	Trichomonad(+)	Candida(+)	The clue cells(+)	Mycoplasma(+)	Chlamydia(+)
CG	8 (8.00)	8 (8.00)	11 (11.00)	0 (0.00)	20 (20.00)
CG HPV(−)	7 (9.49)	4 (5.41)	10 (13.51)	0 (0.00)	14 (18.92)
CG HPV(+)	1 (3.85)	4 (15.38)	1 (3.85)	0 (0.00)	6 (23.08)
LSIL	11 (8.09)	2 (1.47)	25 (18.38)	7 (5.15)	81 (59.56)
HSIL	37 (14.07)	5 (1.90)	68 (25.86)	11 (4.18)	169 (64.26)
CSCC	7 (21.21)	0 (0.00)	9 (27.27)	0 (0.00)	25 (75.76)
*P*	0.0652	0.0200	0.0086	0.0728	0.0000

Data are expressed as n (%)

### Local vaginal immunity factors in the research groups and control group

**Fig. 1:**
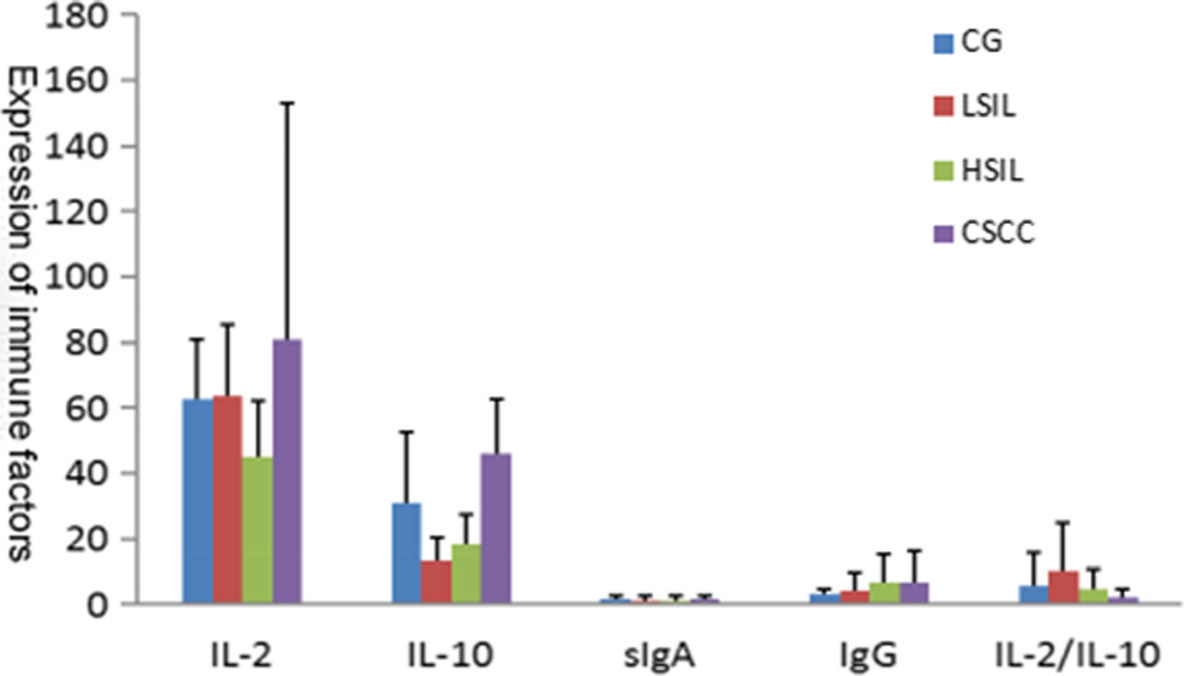
Different level of cervical lesions immune factor content expression differences

The differences in vaginal immune factors among all research groups and the control group were statistically significant (*P* < 0.0001). With the increase in the degree of cervical lesions, IL-2 gradually decreased and subsequently increased in the CSCC group, IL-10 increased, and the Th1/Th2 ratio decreased. Furthermore, SIgA was lower in the cervical lesion group than in the control group, and SIgA increased as cervical lesions progressed. Moreover, IgG was higher in the cervical lesion group than in the control group, and IgG increased as the lesions progressed (Table 4, Fig. 1).

**Table 4:** Differences in the expression of local immune factors in the vagina in the study group and the control group

Group	IL-2^a^ (pg/mL)	IL-10^a^ (ng/L)	sIgA (μg/mL)	IgG^a^ (μg/mL)	IL-2/IL-10^a^
CG	62.45 (18.23)	30.63 (21.96)	1.61 (0.87)	2.92 (1.58)	5.74 (10.22)
CG HPV(−)	64.35 (16.84)	30.78 (22.41)	1.67 (0.94)	2.85 (1.57)	4.91 (5.97)
CG HPV(+)	57.06 (21.15)	30.19 (21.06)	1.41 (0.62)	3.12 (1.62)	8.11 (17.36)
LSIL	63.54 (21.89)	13.41 (6.91)	1.08 (1.35)	3.89 (5.93)	9.97 (14.88)
HSIL	45.09 (17.07)	18.44 (8.86)	1.29 (1.17)	6.62 (8.37)	4.42 (6.24)
CSCC	80.86 (72.05)	45.88 (16.57)	1.42 (1.01)	6.78 (9.59)	2.36 (2.43)
*P*	< 0.0001	< 0.0001	< 0.0001	< 0.0001	< 0.0001

^a^The results among different groups were statistically significant

## Discussion

Cervical lesions include CIN and SCC. A previous study revealed that cervical lesion is an infectious disease caused by HPV [1]. Persistent high-risk HPV infection is a major risk factor to induce cervical lesions and promote its progression [6]. However, the vast majority of HPV infections and CIN I, half of CIN II, and 30% of CIN III cases, can be spontaneously reversed [7], which ultimately does not cause cervical cancer [8]. The cervix is exposed in the vagina, and the local microecology and immunity of the vagina constitute the cervical microenvironment [9]. It remains to be determined whether changes in the cervical microenvironment would affect the infection, pathogenicity and pathological progress of HPV. Stephen Paget has put forward the theory of “seed and soil” as early as 1889, which predicts that as a “seed”, the tumor cell can settle in the “soil” suitable for its growth; that is, tumor cells must cooperate with the surrounding environment to induce the occurrence and development of tumors.

### Vaginal microecology, HPV infection and cervical lesions

Vaginal microecology is an important component of local cervical immunity. Under normal conditions, the dynamic balance system of the vaginal microecology is composed of the microbial flora dominated by dominant bacteria such as Lactobacilli [10, 11]. When this balance is broken, the number of Lactobacilli are reduced or the function of lactobacilli is impaired [12, 13]. This increases the chance of infection of other pathogens, causing the inherent protective mechanism of the vaginal microenvironment to be destroyed [14, 15]. Jinghui Song et al. [16] reported that the decrease in vaginal Lactobacilli and its H_2_O_2_ production in childbearing-age women were related to genital infections.

Compared with the control group, the results of this study revealed that in the research groups, Lactobacilli decreased, H_2_O_2_ function decreased, the density, diversity and normal proportion of flora decreased, the proportions of abnormal flora and the rates of flora imbalance increased, and trichomonad, BV and Chlamydia infections increased; while microecology became imbalanced. So the imbalance was more serious in patients with higher degree of cervical lesions. Lihong Lu [17] reported that the plantation density of vaginal mucosal Lactobacilli decreased in patients with cervical cancer. Roeters [18] reported that the imbalance of vaginal flora could enhance the infection and expression of HPV, and change cervical cytology. This was consistent with the results of this study. It can be inferred that with the increase in the severity of the imbalance of microecology, HPV more easily invaded and pathogenicity was enhanced, inducing the occurrence and progression of cervical lesions. The detection of preformed enzymes revealed that the positive rate of H_2_O_2_ gradually decreased with the worsening of the lesions. The positive rates of SNa, LE, GUS and GADP increased in the CSCC group. This suggests that the vaginal microecological imbalance of CSCC patients was the most serious, and that the imbalance of vaginal microecology and pathogen infections are correlated to the occurrence and development of cervical diseases. The positive rate of H_2_O_2_ in the CSCC group increased, and the reason might be that the number of Lactobacilli significantly decreased in the late stage of the disease, hence H_2_O_2_ production function of the vaginal microbia might compensatorily increase to reduce the local pH. These results suggest that the number of Lactobacilli and H_2_O_2_ function are correlated to high-risk HPV infection and cervical lesions.

This study revealed that no cervical lesions occurred and the evaluation indexes of vaginal microecology were normal in the 26 patients with HPV (+) in the control group. Compared with patients in the research groups and patients with HPV (−) in the control group, these patients had a more normal flora structure, more strong H_2_O_2_ function, the highest rates of density, diversity and normal proportion of flora, and fewer abnormal bacteria and other pathogen infections. It can be speculated that HPV is the main pathogenic factor for cervical lesions, but the predominance of vaginal Lactobacilli, strong H_2_O_2_ function, and vaginal microecological balance induces a very strong inhibition effect on the pathogenicity of HPV infection. Megan A Clarke [19] reported that flora imbalance, bacterial infections and high-risk HPV infection co-promoted the occurrence of lesions and carcinogenesis before cervical cancer. Kriek [20] revealed that chronic inflammatory conditions increased the risk of persistent HPV infection, which was consistent with the results of this study.

Therefore, the vaginal microecological status has an important influence on HPV infection and cervical lesions. Maintaining the vaginal microecological balance and the timely treatment of vaginal infectious diseases can prevent cervical lesions after HPV infection. Increasing the amount of vaginal Lactobacillus and enhancing H_2_O_2_ function can probably improve the outcome of HPV, and slow down and inhibit the occurrence and progression of cervical lesions. The detection of vaginal Lactobacilli, as well as the density and diversity of flora, H_2_O_2_, SNa, LE, GUS and GADP, can be potentially used to monitor and determine the prognosis of cervical lesions and therapeutic effects.

### Local vaginal immunity, HPV infection, and cervical lesions

Local vaginal immune includes the cellular immunity represented by IL-2, IL-10 and humoral immunity mainly mediated by SIgA and IgG [21]. IL-2 is the representative factor of Th1, which can strengthen cellular immunity. IL-10 is the representative factor of Th2, inhibiting immune response. The expression levels of IL-2 and IL-10 and the ratio between the two indirectly reflect the levels of Th1 and Th2 [22]. Under normal conditions, Th1/Th2 keeps in a dynamic balance, and the function of Th1 cells is dominant, to maintain normal immune function of the body. Once the balance is broken, Th1 drifts to Th2, then immunosuppression occurs.

The results of this study revealed that differences in immune factors between the research groups and the control group were statistically significant. With the increase in the degree of cervical lesions, IL-2 increased slightly in LSIL cases, decreased significantly in HSHL cases and increased again in CSCC cases, IL-10 increased with the progression of the disease, and IL-2/IL-10 ratio decreased. Toshiyuki Sasagawa [23] and Peghini [24] reported that after HPV infection, IL-2 decreased and IL-10 increased, which was consistent with part of the results of this study. It is speculated that with the progression of the disease, IL-10 gradually increases and Th1 gradually drifts to Th2, which might be the manifestation of an immune inhibitory state and a further progress of lesions. This may be one of the mechanisms of the immune escape of cervical cancer cells. In this study, although IL-2 increased in CSCC cases, the Th1/Th2 ratio was lowest in CSCC cases, therefore the IL-2/IL-10 drift could better reflect the immune state of the body, and it indicates that the imbalance of T cell immune response was probably more and more obvious with the progression of the disease, causing Th1 to drift to Th2 in the body, and decrease cellular immune function. Hence, the body might gradually fall into an immune inhibitory state, inducing the occurrence and development of cervical lesions after HPV infection. Further studies are needed in the future to support this hypothesis.

SIgA plays an anti-infection role by binding with the microorganisms in the mucosal surface, and neutralizing viruses. This study revealed that SIgA was lower in the research groups than in the control group, but it increased with the progress of cervical lesions. Lilin Yang [25] reported that the secretion of SIgA increased in mild vaginal infection, and decreased in severe infections. Through this study, it was speculated that in early lesions, SIgA participates in local mucosal immunity by binding with corresponding pathogenic microorganisms and neutralizing viruses to prevent pathogens from adhering to the cell surface, allowing it to have a local anti-infection role, and reducing the concentration. In the late stage of the disease, HPV infection persisted, vaginal flora became severely imbalanced, H_2_O_2_-producing Lactobacilli disappeared, and IgA protease secretion decreased; preventing the disulfide bond in hinge region of SIgA from being dissociated [25], and increasing SIgA concentration. Therefore, the duration for SIgA production was short, and the duration of the neutralizing virus was positively correlated with the production of antibodies. Therefore, SIgA level has the potential to be used to immediately evaluate the local vaginal immune and protection function, and predict the direction of the development of lesions.

In this study, IgG was higher in the research groups than in the control group, and significantly increases with the progression of cervical lesions. Shirong Li [26] reported that IgG increased with the progression of lesions. This indicates that with the persistent infection of HPV, a strong and long-lasting humoral immune response is induced in local cervical tissues, producing a large amount of IgG antibodies. This is the reason of the continuous progress of cervical lesions. Therefore, IgG can be used as a monitoring index for determining the condition of the HPV infection and cervical lesions.

Local cervical immune levels significantly changes in patients with cervical lesions. With the increase in the degree of lesions, the levels of IL-10 and IgG increase, and the IL-2/IL-10 ratio seriously shifts, revealing that the body is in an immune inhibitory state that promotes the development of cervical lesions. IL-2 and SIgA levels vary in different stages, and its immediate levels can be used as indexes to predict the progress of the disease and evaluate the prognosis after HPV infection or cervical lesions. Relatively high IL-2 and SIgA levels suggest the normal immune function of the body, but as the disease slowly progresses, IL-2 and SIgA levels are becoming lower, which might reflect that the immunity is weaker and its protective function decreases, therefore promoting disease progression.

## Conclusion

HPV is a “seed” that causes cervical lesions, while vaginal microecology and local immune system are “soil”. The state of the soil determines the “germination and growth” or “death or disappearance” of the seed. Local cervical immune microecological environment plays an important role in the infection and pathogenicity of HPV, and the occurrence and progression of cervical lesions. The reduction of Lactobacillus, or the inhibition of the function of Lactobacillus in the vagina, will lead to the change in the enzymes that are secreted by the microbia, and subsequently cause the changed local environment of the vagina. For example, the reduce in H_2_O_2_ is very obvious when there is a lack of Lactobacillus, and this will cause increased pH in the vagina, which disrupts the protective function of an acidic environment, leading to the propagation of the pernicious bacteria. And then the harmful factors excreted by the pernicious bacteria will affect the normal functions of the cells, and subsequently reduce the immune of the vagina, which will influenced the pathogenicity of HPV infection and the occurrence as well as development of cervical lesions. Therefore, increased vaginal Lactobacillus content and enhanced H_2_O_2_ function, the supplementation of immune factors, and the treatment of other pathogenic bacterial infections to regulate the balance of vaginal microecology and local immunity might contribute to the blocking of HPV infection, and the inhibition of the occurrence and development of cervical lesions. The detection of local cervical microecological and immune factors can help predict HPV infection, provide guidance in the analysis, evaluate prognosis and guide treatment, providing basic research data and new ideas for clinical targeted therapies for cervical lesions.

## Data Availability

The datasets used and/or analysed during the current study are available from the corresponding author on reasonable request.

## Abbreviations

ANOVA: Analyzed by analysis of variance
CIN: Cervical intraepithelial neoplasia
CSCC: Cervical squamous cell carcinoma
ELISA: Enzyme-linked immunosorbent assay
HPV: Human papillomavirus
IgG: Immunoglobulin G
IL-10: Interleukin-10
IL-2: Interleukin-2
SIgA: The concentrations of immunoglobulin A
